# Supplementary material for: Bacillus licheniformis FA6 Affects Zebrafish Lipid Metabolism through Promoting Acetyl-CoA Synthesis and Inhibiting β-Oxidation
Source: Int J Mol Sci. 2022 Dec 30;24(1):673. doi: 10.3390/ijms24010673 (PMC9820476; doi:10.3390/ijms24010673)
Supplement: Supplementary file 1 [file ijms-24-00673-s001.zip › ijms-2089390-supplementary.pdf]

Figure S1: Composition of microbiota communities in the three groups at genus level;

Figure S2: The boxplots of Alpha Diversity Indices. * $p < 0.05$; ** $p < 0.01$;

Figure S3: The boxplots of PerMANOVA analysis result;

Figure S4: Differences between control and HD groups in gut metabolite composition of zebrafish;

Table S1: The detail of differential metabolites;

Table S2: The detail of differential exprssion genes;

Table S3: Ingredient and proximate composition of basal diet;

Table S4: The primers used for RT-qPCR.
