# Supplementary figures and images for: Bacillus licheniformis FA6 Affects Zebrafish Lipid Metabolism through Promoting Acetyl-CoA Synthesis and Inhibiting β-Oxidation
Source: Int J Mol Sci. 2022 Dec 30;24(1):673. doi: 10.3390/ijms24010673 (PMC9820476; doi:10.3390/ijms24010673)

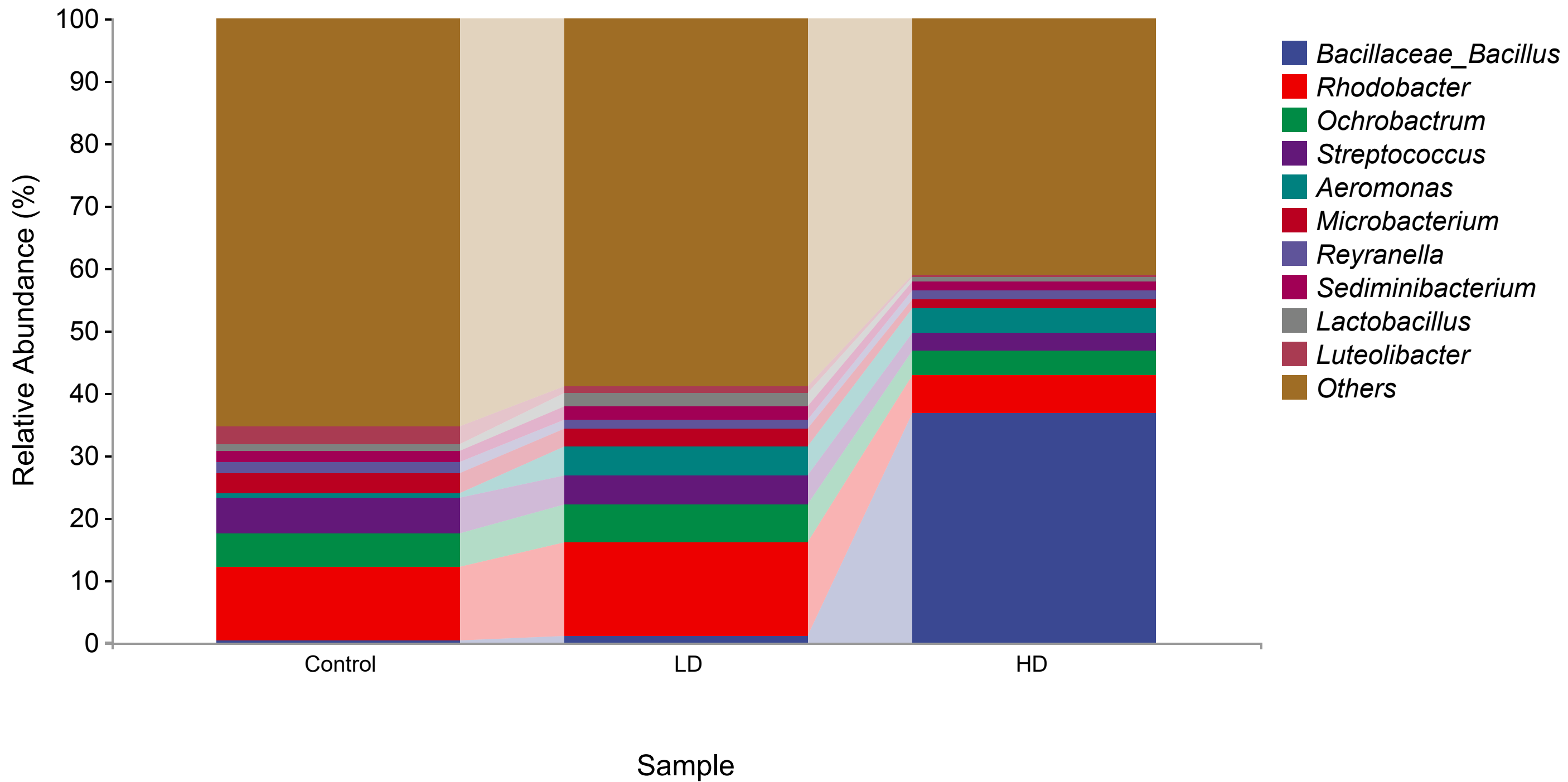

Supplement: Supplementary file 1 [file ijms-24-00673-s001.zip › Figure S1. Composition of microbiota communities in the three groups at genus level.pdf]

# Boxplots of Alpha Diversity Indices

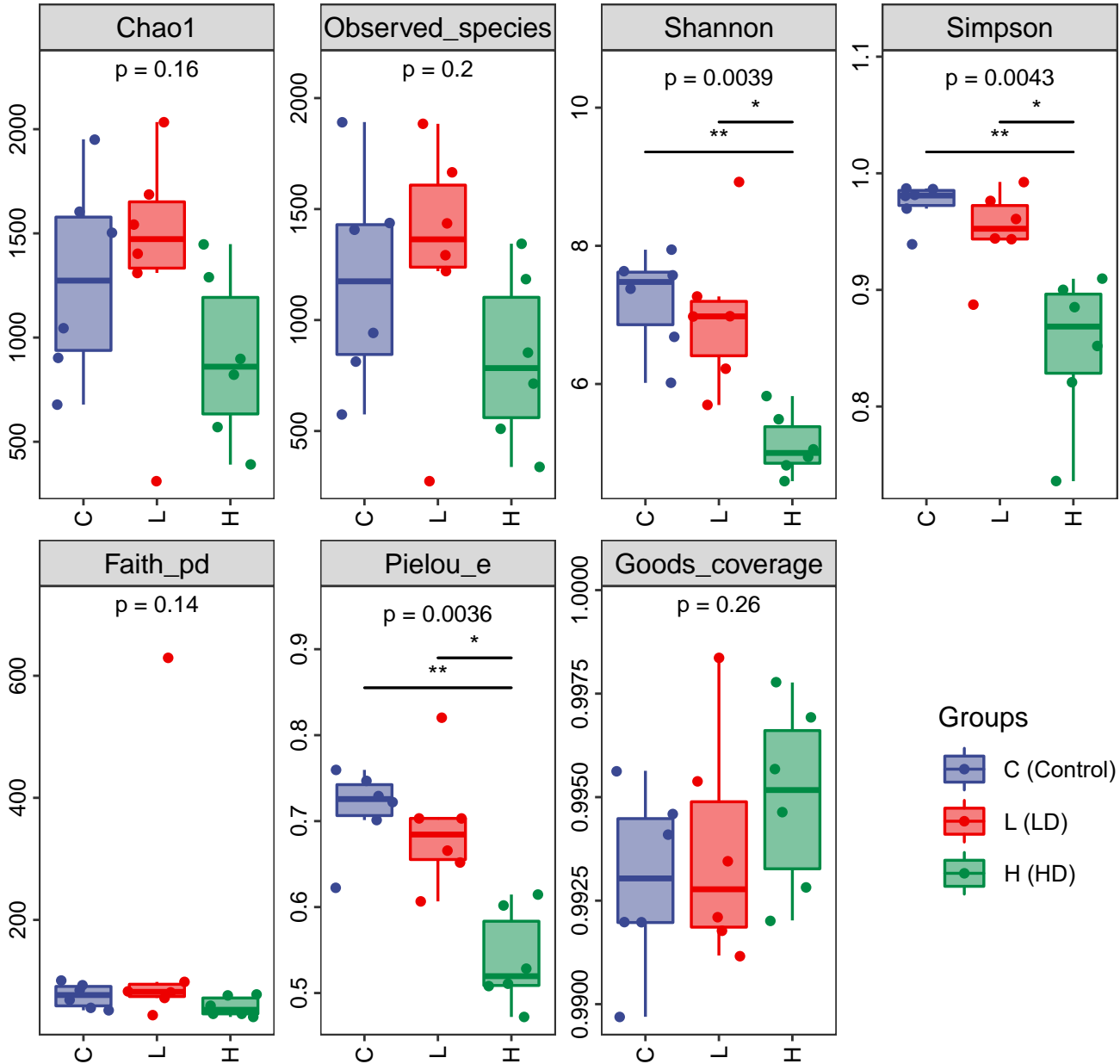

Supplement: Supplementary file 1 [file ijms-24-00673-s001.zip › Figure S2. The boxplots of Alpha Diversity Indices.pdf]

Distances to H

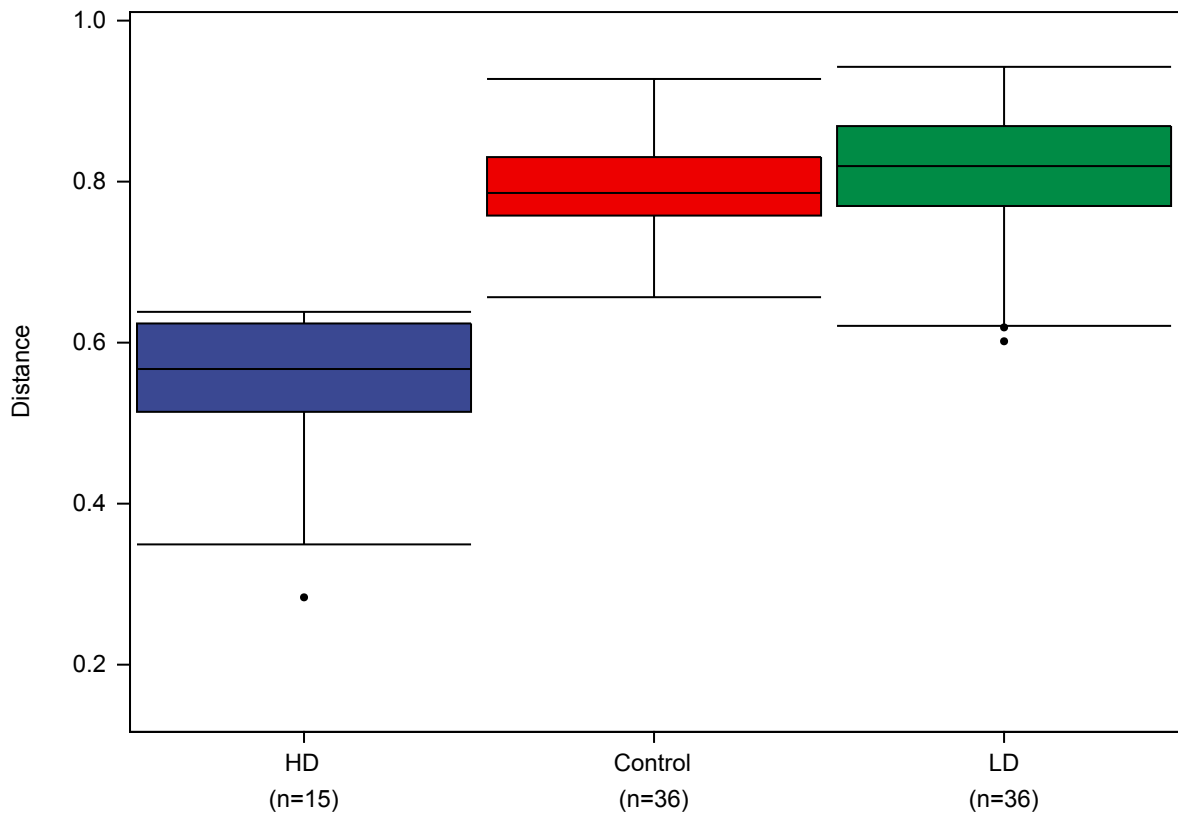

Supplement: Supplementary file 1 [file ijms-24-00673-s001.zip › Figure S3. The boxplots of PerMANOVA analysis result.pdf]

A

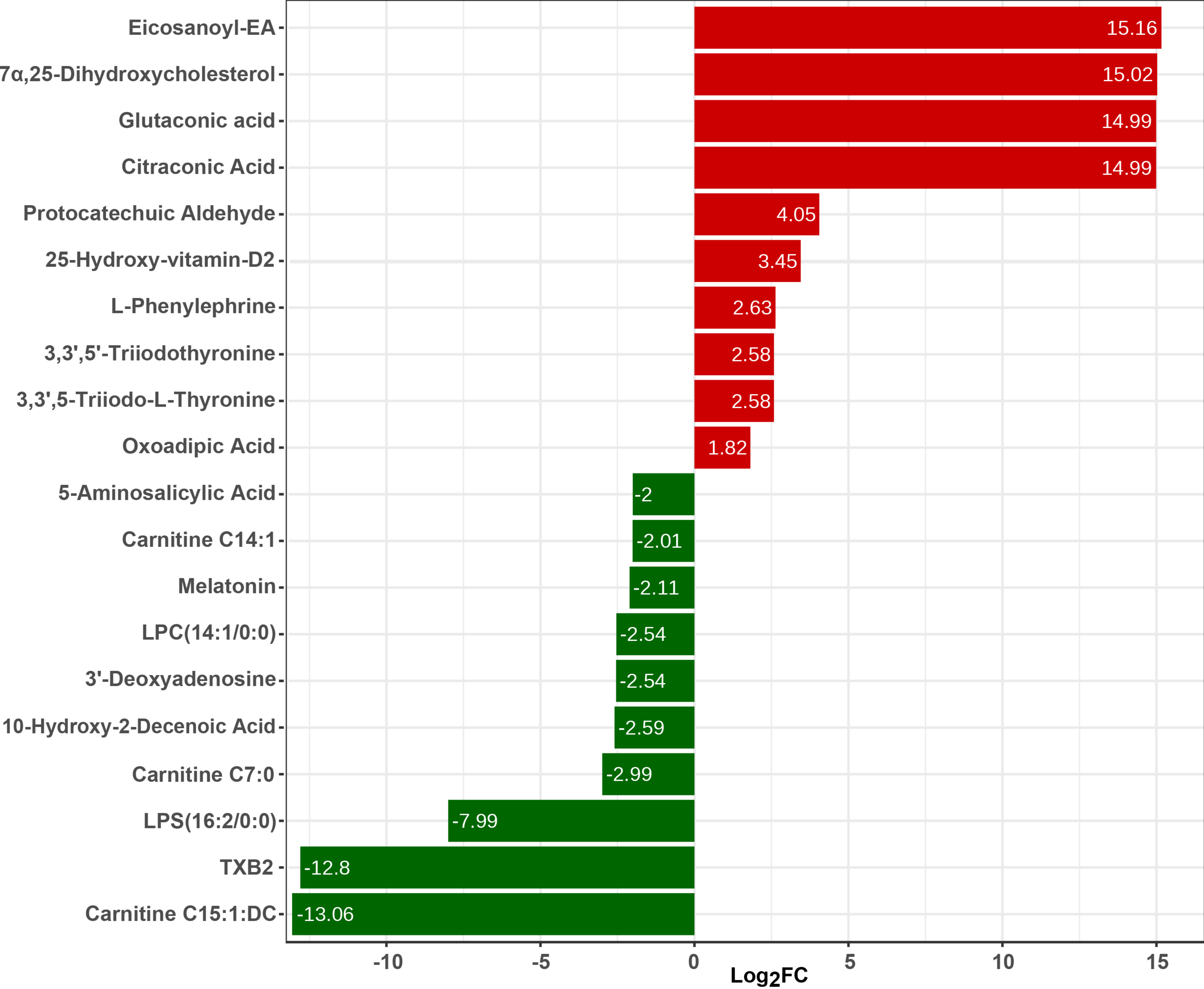

B

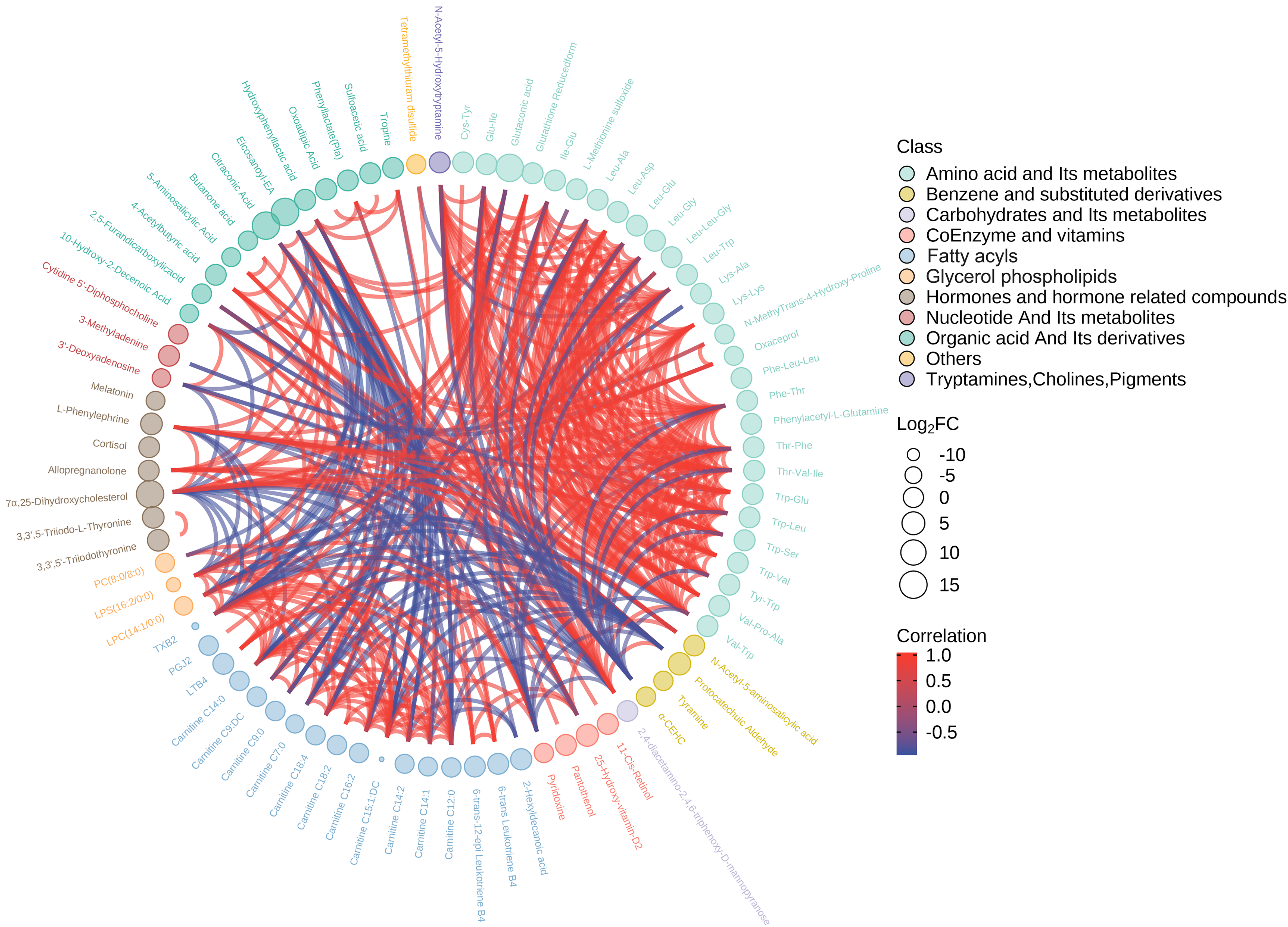

Supplement: Supplementary file 1 [file ijms-24-00673-s001.zip › Figure S4 Differences between control and HD groups in gut metabolite composition of zebrafish.pdf]
